# Treatment modality in type II odontoid fractures defines the outcome in elderly patients

**DOI:** 10.1186/1471-2482-13-54

**Published:** 2013-11-09

**Authors:** Max J Scheyerer, Stefan M Zimmermann, Hans-Peter Simmen, Guido A Wanner, Clément ML Werner

**Affiliations:** 1 Department of Surgery, Division of Trauma Surgery, University Hospital Zurich, Zurich, Switzerland

## Abstract

**Background:**

Odontoid fractures account for approximately 20% of all fractures of the cervical spine. They represent the most common cervical spine injury for patients older than 70 years, the majority being type II fractures (65-74%), which are considered to be relatively unstable. The management of these fractures is controversial. Possible treatment options are either conservative or surgical. Surgical procedures include either anterior screw fixation of the odontoid or posterior C1/C2 fusion. The aim of this study was to compare the outcome of the three treatment modalities in elderly patients.

**Methods:**

Between June 2004 and February 2010, all patients older than 65 years (n = 47) with type II fractures of the odontoid according to the Anderson and D’Alonso classification were retrospectively reviewed.

**Results:**

In the non-operatively managed cohort, 11 patients (79%) died postoperatively within a mean period of 23 months. In all other cases (n = 3), radiographs demonstrated non-union. The mean lateral displacement was 1.9 mm (range 0–5,8 mm) and a mean angulation of 29,1° (range 0-55°) was found.

Anterior screw fixation was carried out in 17 patients. The non-union rate in this cohort was 77%. In patients with a posterior C1-C2 fusion, a bony fusion of the posterior elements was found in 15 of 16 cases (93%). Survival rates were significantly higher among the group of patients who were treated with anterior screw fixation or posterior C1/C2 fusion compared to the conservatively treated group.

**Conclusion:**

We found the best clinical results with low rates of non-union as well as low mortality rates following posterior C1/C2 fusion making this our treatment of choice especially in an elderly patient collective.

## Background

Odontoid fractures account for approximately 20% of all fractures of the cervical spine [1,2]. They represent the most common fractures of the cervical spine for patients older than 70 years and are the most common of all spinal fractures for patients older than 80 years [3]. As a result of demographic developments toward an older population, the incidence of this injury will further increase in the future. The most common fracture types according to the Anderson and d’Alonso classification are type II fractures (65-74%), which are considered to be relatively unstable [4,5]. The fracture occurs at the base of the odontoid between the level of the transverse ligament and the base of the odontoid process.

There is a bimodal population distribution with peaks in early adulthood and in the elderly [6]. In younger patients, these fractures are usually the result of a high-energy trauma resulting from a motor vehicle accident or fall from a substantial height [5]. In older patients, the injury commonly occurs in the setting of a low-energy trauma such as falls from a standing height with hyperextension of the neck resulting in posterior displacement of the odontoid [7].

There is no universally accepted method concerning the management of these fractures, especially in elderly patients [8]. Treatment options for displaced odontoid fractures are either conservative or surgical. Non-operative treatment with a rigid brace may result in fracture healing. However, it is associated with a mortality rate of 26 to 47% in elderly patients [9-11]. Several facts may account for this, including a high rate of non-union, comorbidities and potential fatal complications – especially respiratory-related - due to prolonged external immobilisation. To avoid these problems, surgical procedures providing internal fixation are practiced. Following traditional posterior C1-C2 arthrodesis, union rates between 92,8 and 100% have been described in literature [12,13]. Direct anterior screw fixation has been reported to result in successful fracture healing in around 80% of the cases at least in young patients [14,15].

The aim of this study was to assess the outcome of elderly patients with a type II odontoid fracture depending on the different treatment modalities. In particular, mortality and non-union rate were evaluated.

## Methods

All patients older than 65 years which had sustained a fracture of the odontoid and were initially admitted to the emergency ward of a Trauma 1 centre between June 2004 and February 2010 for assessment were included in this study. Their physical condition preoperatively was defined using the American Society of Anaesthesiologists- (ASA-) classification [16].

Due to the retrospective nature of the study, use of all data exclusively in an anonymized form and the current local regulations, no further approvals of the patient or the local ethics committee were necessary.

Further inclusion criteria were an initial radiography (Multix with an Optitop 150/40/80 tube, Siemens, Munich, Germany; 71–90 kV, 25–40 mAs) or computed tomography (Somatom Definition, Siemens, Munich, Germany; 128-slice dual source CT; 120 kV, 210 mAs) of the cervical spine, both performed in our hospital.

All patients with additional osseous lesions of the cervical spine in the initial radiography apart from the type II odontoid fracture were excluded.

A surgical treatment was proposed to all patients and both possible surgical procedures were explained. The posterior C1-C2 arthrodesis including a screw-rod fixation system according to Harms was the preferred surgical procedure. If a direct anterior screw fixation was elected by the patient, it was performed using one cannulated partially threaded traction screw (36-46 mm, diameter 3,5 mm). For patients who preferred a non-operative treatment, immobilisation in a soft collar orthosis for at least 6 weeks was carried out.

The following parameters were examined retrospectively: gender, age at time of injury, mono- or polytrauma, type of treatment, time between accident and operation. Furthermore, we analysed the mortality rate in each population, as well as the range of motion during follow-up examination and finally fusion rate of the bone.

Within the first year, follow-up examinations were standardised for all patients and took place after 6 weeks, three and 12 months. After that, further consultations were routinely scheduled after two and five years. Additional consultations were scheduled as necessary. Mean follow-up time was 31.1 months (range 1–77). Radiographic analysis during follow-up included lateral and antero-posterior odontoid radiographs. Strict radiographic criteria – such as existence of bony bridges and presence of bony trabeculation were used to identify solid bony fusion. If bony fusion could not be conclusively assessed with radiographs, CT scans were carried out. The latter was the case in 75% of all cases. In patients who had underwent C1-C2 arthrodesis, assessment of bony fusion was related to the posterior structures only.

**Figure 1:**
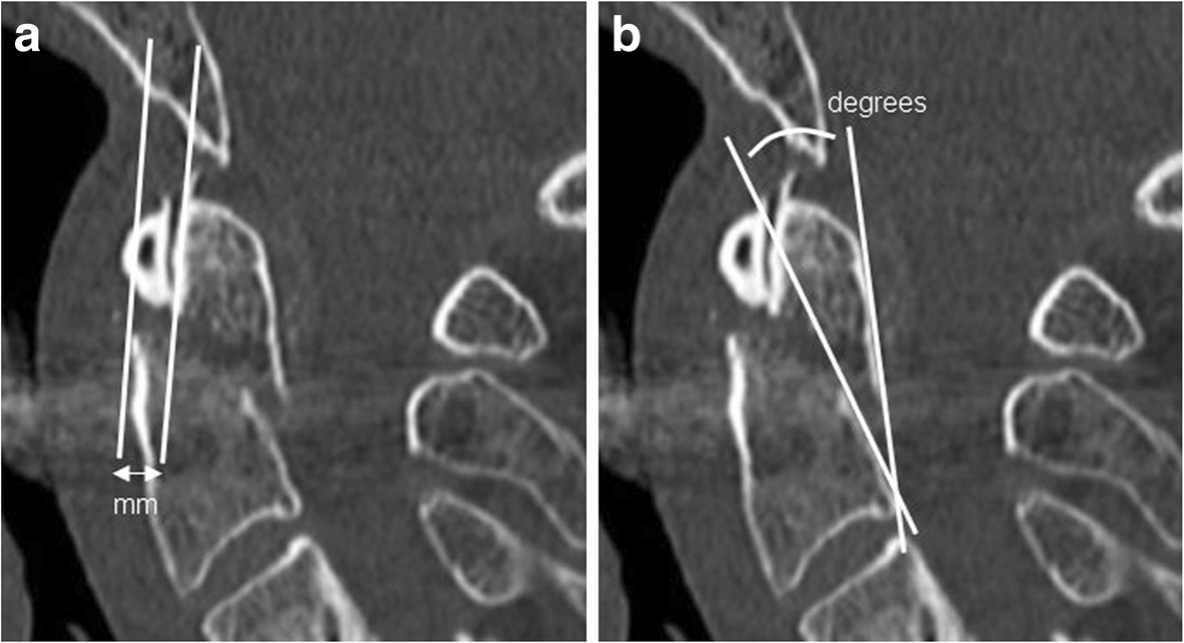
Standard measurement technique of odontoid fracture displacement (a) and degree of fracture angulation (b).

For radiological evaluation of the extent of fracture displacement, a tangent line was drawn along the anterior aspect of the dens and another along the anterior aspect of the body of C2 (Figure 1a). The amount of displacement was then measured at the fracture level by connecting these two lines [17]. The degree of fracture angulation is represented by the angle obtained by placing a tangent line along the posterior aspect of the odontoid and along the posterior aspect of the body of C2 (Figure 1b) [17].

All cervical spine radiographs were retrospectively reviewed by an orthopaedic surgeon and in borderline cases the senior author made a final decision.

A Mann–Whitney- U- Test was performed to evaluate whether there was a benefit in survival after non-operative treatment in comparison to anterior screw fixation or posterior C1-C2 arthrodesis. A probability value ≤ 0.05 was considered statistically significant. Analysis was performed using SPSS1 software (Version 18.0; SPSS Inc, Chicago, IL).

## Results

Between June 2004 and February 2010, forty-seven patients (25 female, 22 male; mean age 81 years) with Anderson and D’Alonso Type II fractures were admitted to our department (Table 1). The mean age in the anterior fixation group was 81 years compared to 82.4 years in the posterior arthrodesis group and 80.1 years in the non-operatively treated cohort. There was a fairly equal distribution of patients in each group; seventeen patients underwent direct anterior screw fixation (8 female, 9 male), in 16 cases posterior atlanto-axial fusion (11 female, 5 male) was performed. The rest (n = 14) were treated with a non-operative management (6 female, 8 male). Preoperative physical condition was assessed using the ASA classification. No significant differences within the cohorts could be found as similar values were observed in all three groups [16]. The mean ASA- score in patients with anterior direct screw fixation was 2,9 (range 2–4), compared to 3,1 (2–5) in those who underwent posterior atlanto-axial fusion, and 3,0 (2–5) in the conservatively treated patient group. Likewise, the proportion of multiple injured patients according to their ISS (≥ 17) was comparable: One patient met this criteria in both the conservatively treated and anterior screw fixation group, whereas there were two multiple injured patients in the posterior fusion group.

**Table 1:** Patient demography

	**Number (w/m)**	**Mean age (years)**	**ASA (range)**	**Died**	**Survival time in month (range)**
Non operativ	14 (6/8)	80.1	3 (2–5)	85%	3 to 54
Anterior screw fixation	17 (8/9)	81	2,9 (2–4)	20%	1 to 28
Posterior C1/2 fusion	16 (11/5)	82.4	3,1 (2–4)	27,7%	21 to 24

In case of anterior screw fixation, all but one of the operations were performed by the same two experienced surgeons using the same technique. All posterior atlantoaxial fusions were performed by three different surgeons.

No surgical complications occurred in the anterior screw fixation collective. The screws had to be exchanged intra-operatively in two cases because they had shown to be a few millimetres too long. In patients with posterior atlantoaxial fusion, bleeding from the venous plexus could be observed in five cases without any clinical consequences. In two cases the screws had to be exchanged intraoperatively and in one patient a reoperation was necessary due to a leakage of the dura mater.

**Figure 2:**
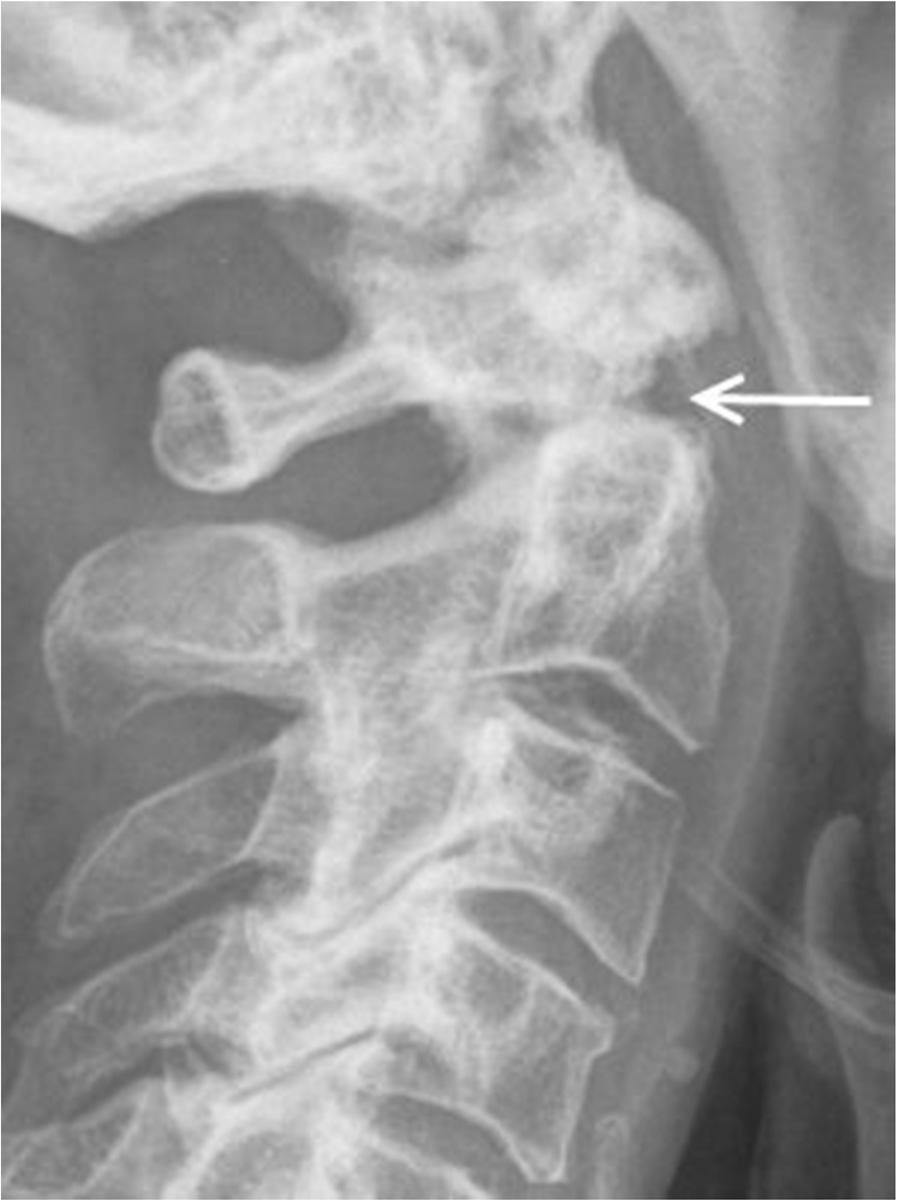
**Lateral radiographs obtained at 3-year follow up in a 77-year-old man who suffered a type II odontoid fracture after a car accident and was treated non-operative.** This image demonstrate lack of fusion.

Patient follow up ranged from 1 to 77 months. In the non-operatively managed cohort, 11 (79%) patients died postoperatively within a mean period of 23 months. Six patients missed the first-year follow-up examination. In all other cases, radiographs demonstrated non-union (Figure 2). The mean lateral displacement was 1.9 mm (range 0–5,8 mm) and a mean angulation of 29,1° was found (range 0-55°). All proximal fragments were tilted in a posterior direction proving hyperextension to be the primary trauma mechanism.

**Figure 3:**
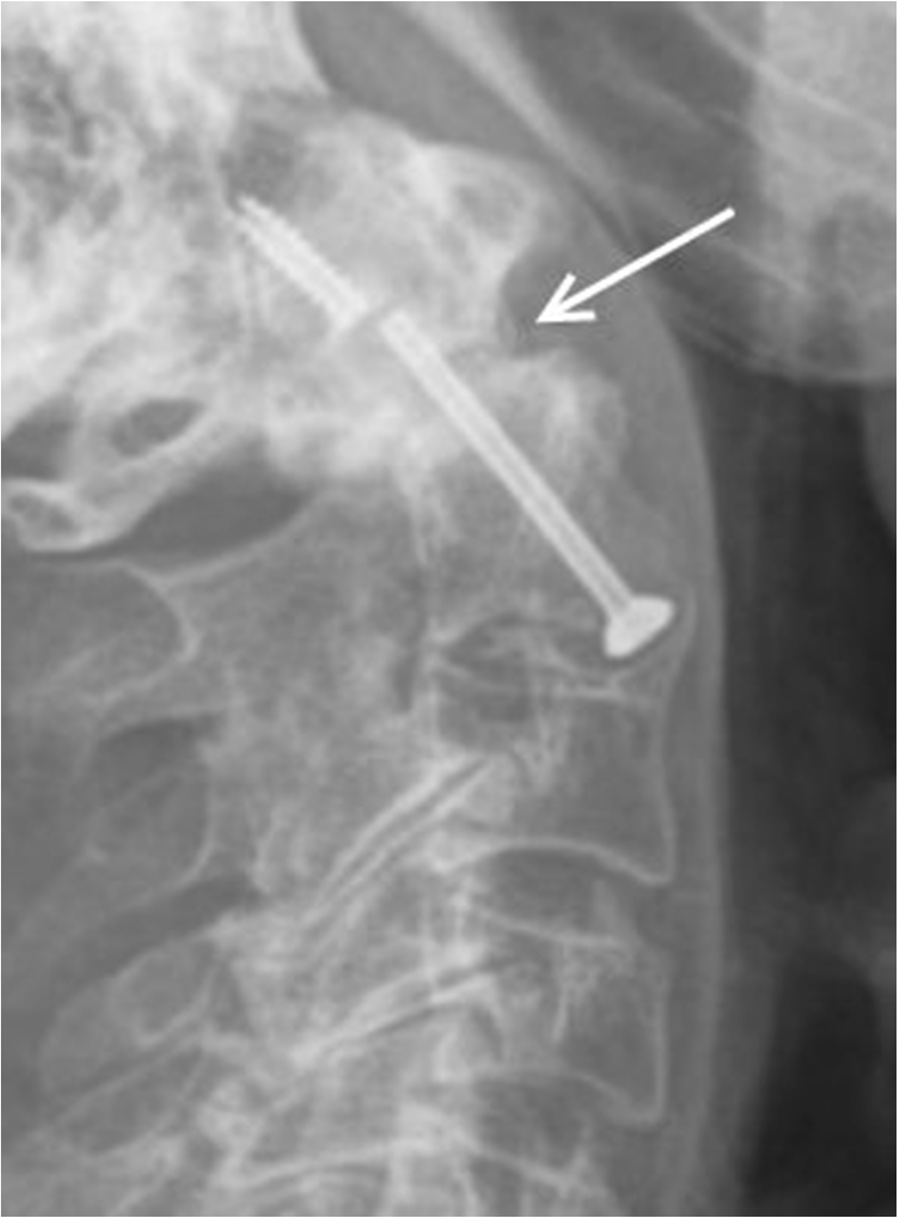
**Lateral radiographs obtained 6 years after anterior screw fixation in a 77-year old man who suffered a type II odontoid fracture after fall of 3 meter.** The image demonstrates a pseudarthrosis at the base of C2 with a breakage of the screw.

**Figure 4:**
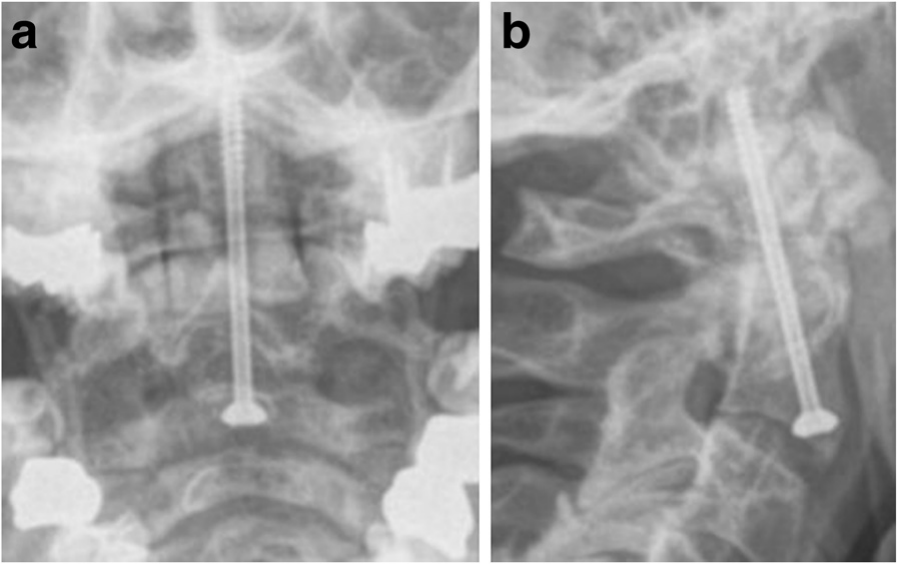
Anterior-posterior (a) and lateral (b) view obtained one year after anterior screw fixation in a 65-year old man who suffered a type II odontoid fracture after collision with a car as a pedestrian.

Anterior screw fixation was carried out in 17 cases. Four people died soon after the hospital discharge and another four missed the re-examination, therefore no radiographs were available in these cases. The non-union rate in the remaining patients was 77%. Delayed-union could be found in 1 case (Figure 3). There was no correlation between non-union and necessity of intra-operative exchange of screws. A solid bony fusion could be identified in two cases (Figure 4). The mean range of motion measured in flexion/extension (chin-sternum distance) was 0,91 cm/12,5 cm, mean rotation to both sides was 37° each, and a mean lateral inclination of 21° was found.

**Figure 5:**
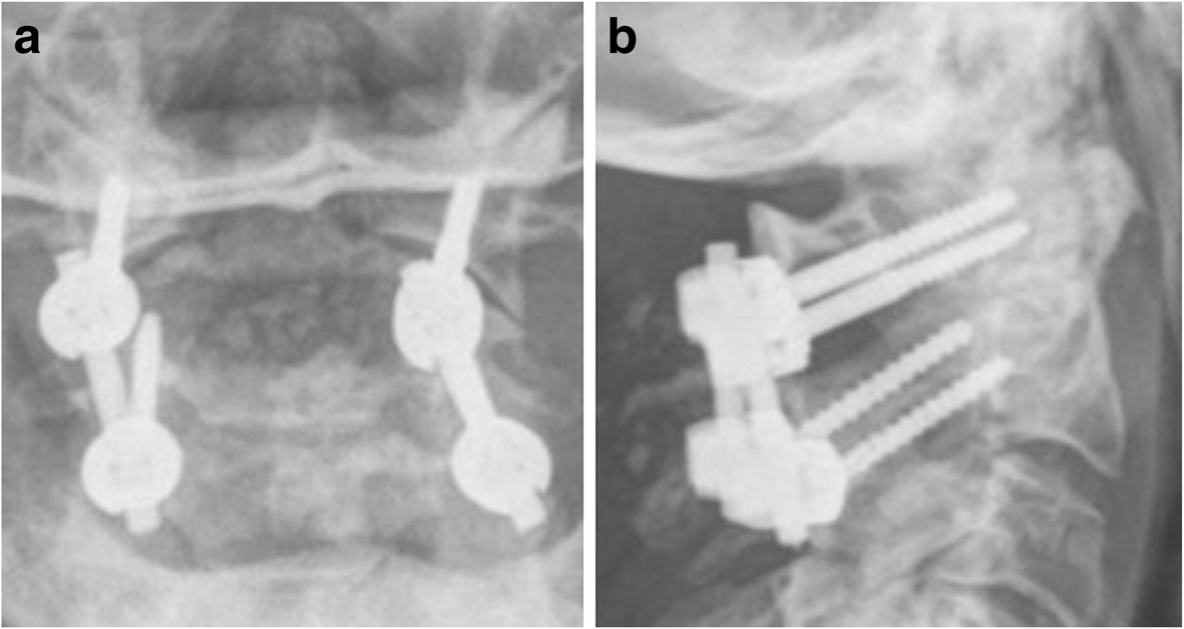
Anterior-posterior (a) and lateral (b) view obtained at 6-months follow-up in a 72-year-old woman who suffered a type II odontoid fracture after a ground level fall and was treated with posterior C1/C2 fusion.

Following posterior C1-C2 fusion, successful bony fusion could be observed in 15 of 16 cases (Figure 5). The mean range of motion for flexion/extension (chin-sternum distance) was 3,8 cm/15,7 cm, mean rotation was 36° to both sides and mean lateral inclination was 19,4°.

With regard to the mortality rate between the three different treatment modalities, our results showed significant differences between conservatively treated patients and patients with anterior screw fixation (p = 0,002) as well as between non-operatively treated patients and patients who underwent posterior C1-C2 fusion (p = 0,009). No statistically significant differences could be observed between the two operatively treated groups (p = 0,696).

## Discussion

Odontoid fractures are the most common injury of the axis – often resulting in atlantoaxial instability. They are especially common in the elderly. The mean age of the patients in our study was 81 years. This correlates well with the results of Ryan and Henderson, who demonstrated that type II fractures according to the Anderson and d’Alonso classification were most common in people aged 70 years and older [18].

In our study, most injuries were attributed to a simple low energy fall.

Contrary to the investigations of Ryan and Taylor, who found a high incidence of concomitant spinal cord injuries especially in the elderly, no neurological deficits could be documented in our study [11]. Other previous investigations are in line with our results showing a low incidence of concomitant neurological deficits [9].

Odontoid fractures – diagnosed as well as missed- pose a difficult clinical problem in elderly patients with an alarming rate of complications, such as respiratory problems, non-union, pain, and death. At the same time, the indication for surgical treatment of odontoid fractures in the elderly as well as choice of procedure remains controversial with no present consensus.

Our clinical study focused on the outcome of patients undergoing external splinting, anterior screw fixation and dorsal C1/C2 fusion. We attempted to elaborate advantages of each treatment modality by evaluating fusion rate, magnitude of fracture angulation and mortality.

In all non-operatively treated patients, the follow up examination showed non-union of the type II fractures (Figure 2). All patients were treated with a cervical orthosis for at least six weeks. One possible cause for the high rate of non-union may be the on-going motion of the cervical spine, even after properly placed external splinting [10]. Residual C1/C2 instability may cause persistent pain, upper extremity paraesthesia and myelopathy [4]. In our opinion, this is therefore an unacceptable result.

Although a posterior displacement of the odontoid could be observed in 62,5% of the non-operatively treated cases, all patients were free of neurologic symptoms. However, there are but a few data in literature describing a relationship between the displacement of the odontoid and myelopathy. For instance, Ryan and Taylor found an incidence of 70% of posterior displacement in patients with myelopathy and Müller et al. mentioned that neurological impairment was associated with posterior displacement of the odontoid in the majority of the cases in their cohort [11,19]. The absence of neurological symptoms in the present study can be explained through Steele’s rule of thirds [20]: The antero-posterior diameter of the ring of the atlas spans approximately 3 cm. The spinal cord and the odontoid process are each approximately 1 cm in diameter; the remaining centimetre of free space allows for some degree of displacement without any neurological sequelae to be expected.

The mortality rate in the non-operatively treated group in our series was 85%, which is substantially higher compared to reports from other clinical series [9-11,21]. However, the observation period in our cohort was quite long and our study focused on the elderly only. Our results are further relativised when considering that Müller et al. demonstrated a 35% in-hospital mortality rate following odontoid fractures in patients aged >70 years [19]. Reasons for the increased mortality in many cases are respiratory-related complications due to prolonged periods of immobilisation.

As mentioned, several options are available for treating odontoid fractures, but currently there is no consensus concerning the best treatment. Surgical options include anterior odontoid screw fixation and posterior atlantoaxial arthrodesis, which is most commonly combined with agglomeration of autologous bone. Both treatment modalities allow early and effective patient mobilisation, which decreases complication rates such as respiratory failure, pneumonia and cardiac arrest [22]. Furthermore, the overall reported high rates of pseudarthrosis with this fracture pattern are substantially lower in the operatively treated groups compared to a non-operatively treated cohort [23].

Anterior direct fracture stabilisation has shown good results in young patients (Figure 4). It has become a popular choice of treatment in noncomminuted fractures that can readily be realigned. The advantage of this procedure is the less traumatic approach allowing rapid postoperative mobilisation. Furthermore, a reduction of morbidity and mortality in comparison to external splinting has been previously extensively investigated and was confirmed by several studies [21,24,25]. In a study conducted by Chiba et al., the authors concluded that anterior screw fixation was the best therapeutic option, but also mentioned the need for bone of decent quality for adequate screw fixation [26]. In a young patient cohort, fusion rates of up to 95% for single screw fixation were found [27]. Significant differences in union rates when using one- versus two-screw fixation techniques in anterior fixation could not be demonstrated in a number of studies [28,29] and satisfying clinical results have been recorded with single screw fixation [30,31]. As mentioned above, anterior fixation was performed by the use of one cannulated screw in our study.

The rate of delayed- or non-union was 77% in our anterior fixation cohort, which is rather high compared to the figures found in literature (Figure 3). However, it is necessary to mention that the patients included in our study were significantly older compared to most studies [27]. Accordingly, the rate of osteoporotic bone was also substantially higher. This is in line with findings in previous investigations, where a high complication rate following anterior fixation could be demonstrated in the elderly [14]. Further causes for these poor results may also be higher rates of comminution at the fracture site or stiffness of the cervical spine preventing ideal positioning of the screw. Furthermore, concomitant thoracic kyphosis or barrel chest deformities make the anatomic reduction difficult in elderly patients. Due to these facts, some authors have come to view this kind of operation as contraindicated in such cases [24].

In contrast to anterior fixation, posterior fusion of C1-C2 results in a high rate of bony union. In a study by Omeis et al., a fusion technique was the treatment of choice and the reported fusion rate was above 90% [13]. Nevertheless, loss of motion at the atlantoaxial joint will follow with this treatment [14] as the high bony fusion rate is achieved at the cost of an almost 50% reduction of cervical rotation and a 10% reduction for flexion and extension [22]. However, the assumption that anterior fixation preserves the atlantoaxial motion is merely theoretical with a reported reduction of up to 50% for C1-2 range of motion following anterior fixation as well [32]. Our results confirm this hypothesis, as the range of motion after posterior fusion and anterior screw fixation was comparable in our study.

Posterior atlanto-axial fusion can be obtained in different ways. We used the screw-rod fixation according to Harms. Correct positioning of the screw-rod system with a complete bony fusion of the posterior structures could be observed in all of the follow-up patients with a posterior C1-C2 fusion. This is in accordance with recent literature demonstrating healing rates of around 93% following posterior atlantoaxial fusion [5,33]. The advantage of this method is the ability to achieve stability without need for prior anatomical reduction of the atlantoaxial articulation. Therefore, thoracic spine and chest deformities in elderly patients have no further negative impact. Furthermore, posterior fusion can also be performed as a salvage operation when anterior screw fixation has failed.

We acknowledge several limitations of the present study. First, due to the retrospective study design we were dependant on complete and accurate patient medical charts to evaluate the physical condition on admission. However, although data collection was performed in a routine setting by trained personal of the Trauma center, we could not ensure with final certainty the completeness of our data. Second, the study was conducted at a single designated trauma centre without randomisation making comparison of the different groups difficult. However, physical conditions were comparable in all three groups. Finally, the relatively low sample size makes further investigation necessary in order to confirm the clear trend demonstrated in our present study and to optimise the statistical power.

## Conclusion

In general, odontoid fractures are injuries that occur during the last years of life. On the basis of our findings and with regard to the current literature, we concur with other authors who recommend surgery for all acute type II fractures and argue for early surgical treatment especially in elderly patients. Consecutive complications resulting from pseudarthrosis and rigid bracing can thereby be avoided. In our opinion, only posterior atlantoaxial fusion techniques led to acceptable healing rates. The range of motion was comparable to anterior screw fixation. Concerning bone quality, the posterior technique according to Harms is our method of choice in elderly patients. Furthermore, we demonstrated good clinical results as well as reduced mortality in comparison to conservatively treated patients.

## Competing interests

The authors confirm that there are no conflicts of interest, whether financial or different nature.

## Authors’ contributions

MJS: Study conception and design; Acquisition of data; Analysis and interpretation of data; Drafting of manuscript. SMZ: Analysis and interpretation of data; Drafting of manuscript; Proofreading and revision as native English speaker. HPS: Study conception and design; Critical revision. GAW: Study conception and design; Critical revision. CMLW: Study conception and design; Analysis and interpretation of data; Critical revision. All authors read and approved the final manuscript.

## Pre-publication history

The pre-publication history for this paper can be accessed here:

http://www.biomedcentral.com/1471-2482/13/54/prepub
